# Editorial Board Members’ Collection Series: Biomimetic Design, Constructions and Devices in Times of Change I

**DOI:** 10.3390/biomimetics9100614

**Published:** 2024-10-10

**Authors:** Ille C. Gebeshuber

**Affiliations:** Institute of Applied Physics, TU Wien, 1040 Vienna, Austria; gebeshuber@iap.tuwien.ac.at; Tel.: +43-1-58801-13483

**Keywords:** biomimetic design, adaptive structures, health diagnostics, ergonomics, sustainable materials, peptide-based detection, prosthetic feet, agricultural efficiency, biodegradable materials, COVID-19 diagnostics

## Abstract

In light of recent global crises, including climate change, species extinction, the COVID-19 pandemic, social upheavals and energy supply challenges, this Special Issue of *Biomimetics*, entitled “Editorial Board Members’ Collection Series: Biomimetic Design, Constructions and Devices in Times of Change”, aims to explore innovative solutions through biomimetics. This collection features research on various biomimetic applications, such as the peptide-based detection of SARS-CoV-2 antibodies, ergonomic improvements for prolonged sitting, biomimicry industry trends, prosthetic foot functionality and agricultural machinery efficiency. The methods employed include peptide synthesis for diagnostics, simulation software for ergonomic designs, patent analysis for biomimicry trends and engineering discrete element methods for agricultural applications. The findings highlight significant advancements in health diagnostics, ergonomic safety, technological development, prosthetics and sustainable agriculture. The research underscores the potential of biomimetic approaches to address contemporary challenges by leveraging nature-inspired designs and processes. These insights contribute to a broader understanding of how biomimetic principles can lead to adaptive and sustainable solutions in times of change, promoting resilience and innovation across various fields.

## 1. Introduction

In recent times, the world has faced multiple crises, including climate change, species extinction, the COVID-19 pandemic, social upheavals and energy supply challenges. These crises demand innovative solutions that can adapt to changing conditions. The Special Issue entitled “Editorial Board Members’ Collection Series: Biomimetic Design, Constructions, and Devices in Times of Change” [1] in the journal *Biomimetics* aims to provide a platform for such innovations by bringing together research that leverages the principles of biomimetics. This editorial presents the papers included in this Special Issue, discusses their contributions to the field and places them within the broader context of current global challenges.

## 2. Overview of Published Papers

The paper entitled “Novel Peptide-Based Detection of SARS-CoV-2 Antibodies” [2] by authors Aliye Bulut, Betul Z. Temur, Ceyhun E. Kirimli, Ozgul Gok, Bertan K. Balcioglu, Hasan U. Ozturk, Neval Y. Uyar, Zeynep Kanlidere, Tanil Kocagoz and Ozge Can reports on the development of peptides that mimic the receptor-binding domain of the SARS-CoV-2 spike protein for the detection of antibodies. The peptides were tested using a quartz crystal microbalance (QCM) sensor, showing potential for rapid and efficient diagnostic applications, particularly during pandemics.

In the paper entitled “Designing and Simulation Assessment of a Chair Attachment Air Blowing Methods to Enhance the Safety of Prolonged Sitting” [3], the authors Mahmoud Z. Mistarihi, Ammar A. Al-Omari and Abdullah F. Al-Dwairi propose a novel chair attachment design with an optimal air-blowing technique to reduce the negative effects of prolonged sitting, such as musculoskeletal disorders. The design was validated using simulation software, showing promising results for ergonomic improvement and safety.

In “Biomimicry Industry and Patent Trends” [4], Haejin Bae examines the technological and industrial trends in biomimicry through patent analysis. The study reveals that biomimicry technology is in a growth phase, with significant contributions from Korea and the United States. The article highlights the potential for future developments in this field.

“The Moment Criterion of Anthropomorphicity of Prosthetic Feet as a Potential Predictor of Their Functionality for Transtibial Amputees” [5] by Mark Pitkin introduces the Index of Anthropomorphicity (IA) as a new parameter for evaluating the functionality of prosthetic feet. The IA correlates with patient comfort and gait characteristics, providing a valuable tool for improving prosthetic design and selection.

The research published with the title “Simulation and Structural Analysis of a Flexible Coupling Bionic Desorption Mechanism Based on the Engineering Discrete Element Method” [6] by authors Jinguang Li, Hongyan Qi, Yunhai Ma, Peng Gao and Baoguang Wu focuses on a bionic desorption mechanism inspired by the sandfish (*Scincus scincus*) for reducing soil adhesion in agricultural machinery. The study uses the engineering discrete element method (EDEM) to simulate soil interaction, showing effective drag reduction and anti-adhesion properties.

## 3. Contextualizing the Research in Current Global Challenges

The contributions to this Special Issue are particularly relevant in the context of today’s global challenges. Each paper advances the field of biomimetics and addresses specific issues related to health, ergonomics and human well-being, technology development, prosthetics and agricultural efficiency.

### 3.1. Health and Medicine

The field of health and medicine is crucial for improving human life expectancy and quality of life, yet it faces significant global challenges. With the rising prevalence of chronic diseases such as diabetes, cardiovascular diseases and cancer, researchers are focusing on developing advanced diagnostic tools, personalized medicine and innovative treatment options [7]. Efforts are being made to explore gene editing technologies, such as CRISPR [8], and their potential applications in treating genetic disorders. Additionally, the ongoing COVID-19 pandemic has underscored the importance of rapid vaccine development and global health preparedness, highlighting the need for continued research and international collaboration in infectious disease control and prevention.

The peptide-based detection of SARS-CoV-2 antibodies by Bulut et al. [2] exemplifies the rapid response required in health crises like the COVID-19 pandemic. By developing efficient diagnostic tools, the research contributes to the timely identification and management of infectious diseases, showcasing the potential of biomimetic approaches in medical diagnostics.

### 3.2. Ergonomics and Safety

Ergonomics and safety are essential for optimizing human performance and well-being in various environments, particularly in the workplace. The shift towards remote work and increased automation presents new challenges in maintaining ergonomic standards and ensuring safety in human–robot interactions [9]. Research is focused on developing guidelines and technologies that can adapt to these changing conditions [10]. Effective ergonomic interventions are necessary to prevent musculoskeletal disorders and enhance productivity, while ensuring that the integration of automation and robotics in the workplace does not compromise human safety.

Mistarihi et al.’s work on improving the safety of prolonged sitting [3] addresses the growing concern regarding sedentary lifestyles and their health implications. The innovative chair attachment design offers a practical solution to mitigate musculoskeletal disorders, enhancing the quality of life for individuals in various settings, from office environments to home use.

### 3.3. Biomimetics

Biomimetics involves the study and imitation of nature’s designs and processes to develop innovative materials and technologies. This field faces the challenge of translating complex biological systems into practical and hopefully sustainable [11] engineering solutions. Efforts in creating bio-inspired materials and soft robotics that mimic natural organisms are pioneering and transformative [12]. The potential applications of biomimetics in medicine, such as developing prosthetics that emulate the human hand or creating materials that replicate the properties of human skin, are vast and transformative. However, scalability and ethical considerations remain critical issues that must be addressed.

Bae’s analysis of biomimicry industry and patent trends [4] provides valuable insights into the technological advancements and economic potential of biomimetic technologies. The growth phase of biomimicry highlighted in the paper underscores the increasing recognition of nature-inspired solutions in addressing complex engineering and environmental problems.

### 3.4. Materials Science

Materials science is dedicated to the discovery and design of new materials that can meet the demands of modern technology and sustainability. Researchers are leading efforts to develop advanced materials with enhanced properties, such as lightweight composites for aerospace applications [13] and high-capacity batteries for energy storage [14]. The challenge lies in creating materials that are not only high-performing but also environmentally sustainable [15]. Efforts are being made to improve recycling techniques and develop biodegradable materials to reduce the environmental impact of material production and disposal.

Pitkin’s introduction of the Index of Anthropomorphicity (IA) for prosthetic feet [5] addresses the critical need for better prosthetic design. By providing a quantifiable measure of prosthetic functionality, the research facilitates the development of more comfortable and effective prostheses, thereby improving the lives of amputees.

### 3.5. Sustainability

Sustainability research aims to develop solutions that balance economic development, environmental protection and social well-being. The urgent challenge presented by climate change necessitates innovative approaches to reduce greenhouse gas emissions, promote renewable energy sources and implement sustainable agricultural practices. Researchers are working on developing technologies and policies that promote sustainable energy and resource management. The transition to a sustainable future requires global cooperation and the implementation of practices that can be adopted at both local and international levels [16].

The flexible coupling bionic desorption mechanism developed by Li et al. [6] represents a significant advancement in agricultural technology. By reducing soil adhesion, the mechanism enhances the efficiency of agricultural machinery, contributing to more resilient and productive farming practices.

### 3.6. Engineering and Design

Engineering and design are critical for addressing complex global challenges through innovative solutions. Researchers are pushing the boundaries of what is possible, from developing resilient infrastructure to withstand the impacts of climate change to integrating artificial intelligence in engineering design. The challenge is to innovate responsibly, ensuring that new technologies and designs consider ethical implications and societal impacts [17]. Sustainable engineering practices and the development of technologies that enhance human capabilities without compromising safety or ethical standards are essential for future progress.

## 4. Integrating Insights from Our Previous Work

In our previous work, “Status and Perspectives of Commercial Aircraft Morphing” [18] and “Engineered Materials: Bioinspired ‘Good Enough’ versus Maximized Performance” [15], we have explored the application of biomimetic principles in different contexts. The first paper discusses the challenges and future prospects of morphing systems in commercial aircraft, emphasizing the need for adaptive structures and control systems. The second paper advocates for the adoption of engineered living materials (ELMs) that prioritize sustainability and recyclability, drawing inspiration from nature’s resource efficiency.

The research on commercial aircraft morphing systems aligns with the broader theme of adapting to change. Just as morphing wings can adjust to different flight conditions, biomimetic designs in other fields must also be flexible and responsive to dynamic environments. The advancements in adaptive structures and control systems discussed in our paper can inform the development of more resilient and versatile biomimetic technologies.

Our work on engineered living materials highlights the importance of sustainability in material design. The principle of “good enough” performance, as opposed to maximized performance, encourages the development of materials that are easier to recycle and decompose. This approach is crucial for addressing environmental challenges associated with material utilization and aligns with the sustainable innovations presented in the current Special Issue.

## 5. Conclusions

The papers in this Special Issue of *Biomimetics* showcase the diverse applications of biomimetic principles in addressing contemporary challenges. From health diagnostics and ergonomic improvements to technological advancements and agricultural efficiency, the research contributions provide valuable insights and practical solutions inspired by nature. As we continue to face multiple global crises, the integration of biomimetic design, constructions and devices offers a promising path toward sustainable and adaptive solutions (see Table 1).

By drawing on the lessons from nature and leveraging interdisciplinary research, we can develop innovative technologies that have the potential to solve current problems and perhaps even pave the way for a more resilient and sustainable future. The insights from our previous work further reinforce the importance of adaptive and sustainable approaches in biomimetic research, underscoring the potential of nature-inspired solutions in times of change.

Prof. Dr. Ille C. Gebeshuber

Dr. Antonio Concilio

Guest Editors

## Figures and Tables

**Table 1:** Interconnections between selected global challenges and specific research in this Special Issue aimed at addressing them.

Category	Global Challenges	Research Needs
Health and Medicine	SARS-CoV-2 [2]	Peptide mimetics [2], biosensor [2], antibody detection [2]
	Excessive sitting [3]	Ergonomics [3], musculoskeletal disorders [3]
Ergonomics and Safety	Excessive sitting, musculoskeletal disorders [3]	Design requirements [5], moment criterion of anthropomorphicity [5]
Biomimetics	Sustainability [15], recycling [15]	Biomimetic technology [4], bionic non-smooth surface [6], sandfish (*Scincus scincus*) [6], wedged structure [16], serrated structure [6], flexible desorption [6]
Materials Science	High-performance materials, limb prosthetics [5]	Engineered living materials (ELMs) [15], biomimicry trend [4], biological characteristics [3], quartz crystal microbalance [2]
Sustainability	Sustainability [14], recycling [14]	Engineered living materials (ELMs) [14], bio-inspired design [15]
Engineering and Design	Stress concentration, control systems	Adaptive structures [18], morphing wings [18], embedded kinematics [18], distributed actuator and sensor networks [18], engineering characteristics [4], patent technology [4]
